# Variation of River Islands around a Large City along the Yangtze River from Satellite Remote Sensing Images

**DOI:** 10.3390/s17102213

**Published:** 2017-09-27

**Authors:** Haiyun Shi, Chao Gao, Changming Dong, Changshui Xia, Guanglai Xu

**Affiliations:** 1 School of Marine Sciences, Nanjing University of Information Science & Technology, Nanjing 210044, China; shihaiyun14@163.com; 2 Department of Geography & Spatial Information Techniques, Ningbo University, Ningbo 315211, China; gaoqinchao1@163.com; 3 Department of Atmospheric and Oceanic Sciences, University of California, Los Angeles, CA 90095, USA; 4 Key Lab of Marine Science and Numerical Modeling, First Institute of Oceanography, State Oceanic Administration, Qingdao 266061, China; xiacs@fio.org.cn; 5 Laboratory for Regional Oceanography and Numerical Modeling, Qingdao National Laboratory for Marine Science and Technology, Qingdao 266237, China; 6 College of Territorial Resources and Tourism, Anhui Normal University, Wuhu 241000, China; guanglaixu@163.com

**Keywords:** flow and sediment, Nanjing, river islands, remote sensing, soil erosion, sensor, the Yangtze River

## Abstract

River islands are sandbars formed by scouring and silting. Their evolution is affected by several factors, among which are runoff and sediment discharge. The spatial-temporal evolution of seven river islands in the Nanjing Section of the Yangtze River of China was examined using TM (Thematic Mapper) and ETM (Enhanced Thematic Mapper)+ images from 1985 to 2015 at five year intervals. The following approaches were applied in this study: the threshold value method, binarization model, image registration, image cropping, convolution and cluster analysis. Annual runoff and sediment discharge data as measured at the Datong hydrological station upstream of Nanjing section were also used to determine the roles and impacts of various factors. The results indicated that: (1) TM/ETM+ images met the criteria of information extraction of river islands; (2) generally, the total area of these islands in this section and their changing rate decreased over time; (3) sediment and river discharge were the most significant factors in island evolution. They directly affect river islands through silting or erosion. Additionally, anthropocentric influences could play increasingly important roles.

## 1. Introduction

River islands are structural sediment deposits which extend above the water level in braided streams. They divide a river into multiple channels and form the connection of interrelation and interaction between two channels [1,2]. Riverine islands have various shapes and wildly different surface areas. They are more stable than central bars and shoals. Generally, the head of a river island is greatly affected by wave action resulting in erosion. Sediment deposition occurs at its tail where shoals are born. Consequently, the island continuously crawls along the river channel, becoming longer and narrower in the process. This process is dependent on sediment flow and boundary conditions.

River island evolution has an important influence on the river channel, the river bed, embankments, ports, and ecosystems, biological communities, human activities on the island itself. Thus, the study of river islands has important real-world applications [3]

Previous research on the shifting, collapse and erosion of river islands found that these phenomena significantly affect river channels, island bodies and banks [4,5,6,7,8]. Some scholars studied the area changes of river islands. Li et al. [9] analyzed river islands in the middle and lower reaches of Yangtze River quantitatively from Landsat and Google Earth images and found that the area of these islands decreased due to the reduction of sediment availability after the Three Gorges Reservoir began to fill. Gao [10] analyzed eight river islands in Mawutong section of Yangtze River and found that their areas firstly increased then decreased over 30 years. The extraction methods of surface features were also been discussed. Chen et al. [11] extracted water information of Shangri-la County’s wetland from ETM+ images using threshold value method, interpolation method and spectral relationship method and found that threshold value method cannot distinguish the mountain shadow and water easily. Some other scholars analyzed the influencing factors including sediment in the rivers. Wang et al. [12] discussed sediment yield, transport and deposition by the sediment budget method, finding that 10% of fine sediment transport by the Yangtze River estuary is done by tidal flow into the sea. Sediment supply is a significant determinant of channel formation and maintenance [13]. Park and Latrubesse [14] demonstrated their MODIS (Moderate Resolution Imaging Spectroradiometer)-based model in capturing the spatial and temporal variability of surface sediments in the Amazon River Basin, the largest river system on Earth.

With the development of remote sensing (RS) technology, the temporal and spatial resolution of RS images have continually improved. More remotely sensed data (MODIS, TM (Thematic Mapper), ETM (Enhanced Thematic Mapper)+, Quickbird, radar, aerial photo etc.) are being applied in various geoscience research field, including, but not limited to, land resources planning and environmental monitoring [15,16]. The boundary between land and water can be easily extracted using remote sensing techniques because of its macroscopic characteristics [17]. Based on time span and spatial resolution requirements, this study utilized and analyzed Landsat5 TM and Landsat7 ETM+ derived images of the study area.

However, few studies on the quantitative information extraction and influencing factor analysis of river islands have been conducted [9,10]. In this paper, the evolution of seven typical river islands in study area was quantitatively evaluated based on remotely sensed TM and ETM+ data. The primary factors were identified by combining annual runoff and sediment discharge data as measured at the hydrological station. The primary objective was to reveal the spatial and temporal variation of river islands, and to evaluate the factors influencing this variation.

## 2. Study Area and Data

### 2.1. Study Area

The Yangtze River (also known as the Changjiang River) is the world’s third longest river and the longest one in Asia. It flows from Anhui Province to Jiangsu Province. The Nanjing section is located in its lower reaches, between 118°22′–119°14′ and 31°14′–32°37′, for a total river length section of 95 km. The Yangtze River flows through central Nanjing from the southwest to northeast. Nanjing, which features a northern, subtropical monsoonal climate, lies in southeast of Jiangsu Province and the mean annual air temperature is 15.7 °C. Annual precipitation is 1106.5 mm [18]. The Qinhuai River and Chuhe River located south and north of Yangtze River, respectively, are two primary tributaries which flow through Nanjing. Most of the land along the river is classified as plains, specifically valley plains and lakeshore plains, where the agro type is dominated by yellow brown soil. The lower reaches of the Yangtze River in the Nanjing region, play host to many riverine islands. Because of sediment transport and deposition, tidal backwater and river tides, more than a dozen river islands with a variety of shapes and sizes appear in Nanjing section of Yangtze River (hereinafter referred to as NYR). They are shoals, washland, shallows and river islands, and their average elevation is four to seven meters.

**Figure 1:**
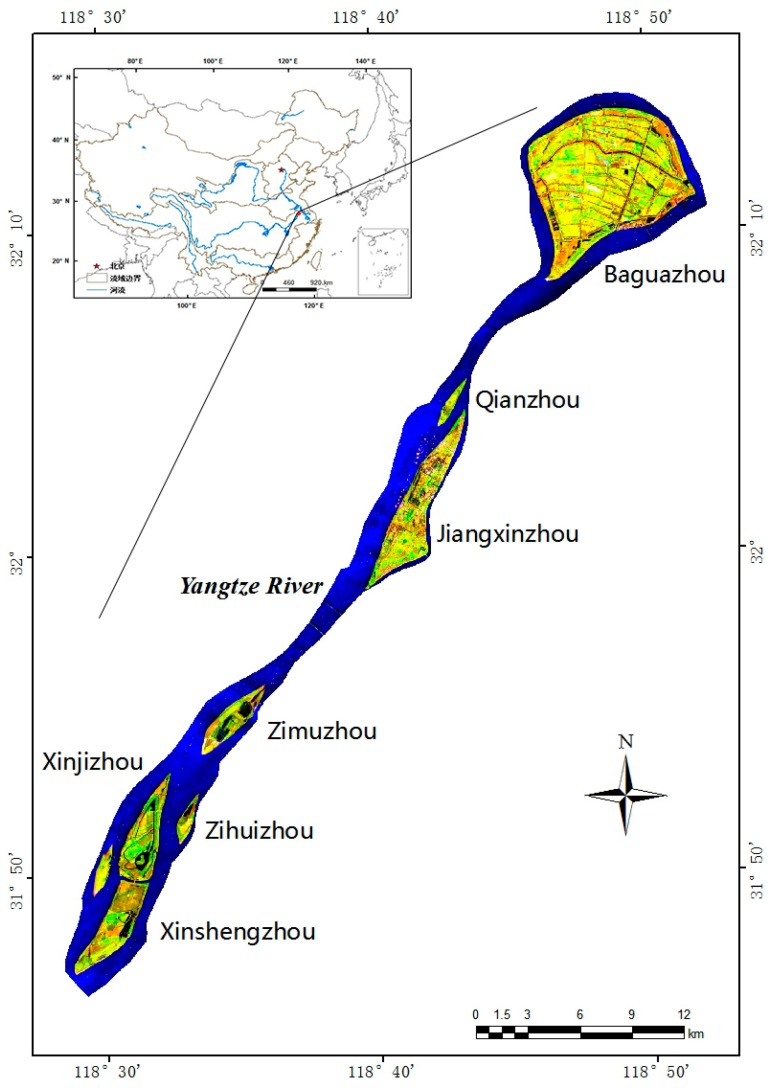
Map of the Nanjing Section of the Yangtze River, showing the river islands Xinshengzhou, Xinjizhou, Zihuizhou, Zimuzhou, Jiangxinzhou, Qianzhou and Baguazhou by Landsat7 ETM+ acquired on 12 October 2015.

The seven selected river islands, from south to north are Xinshengzhou, Xinjizhou, Zihuizhou, Zimuzhou, Jiangxinzhou, Qianzhou and Baguazhou (Figure 1). The first six islands are long and narrow, while Baguazhou island is located at the river’s inflexion point, where the flow of the Yangtze River turns east from the northeast. Baguazhou is the largest river island of the group, and third largest within the river. It plays host to advanced agriculture and a population of approximately thirty-three thousand and is shaped like a cattail leaf fan. Xinjizhou and Xinshengzhou islands were originally linked, but became segmented after a flood in 1998. The changes in these seven river islands are expected to reflect changes in other islands in the Nanjing section and even in the lower reaches of Yangtze River effectively and provide basis to support healthy managerial practices in the river channel and bank.

### 2.2. Data

#### 2.2.1. Remotely Sensed Data

To study the spatial-temporal characteristics of these islands, Landsat TM (Thematic Mapper) and ETM+ (Enhanced Thematic Mapper) data from 1985 to 2015, was acquired at five-year intervals (Table 1). The dataset was provided by Geospatial Data Cloud at the Computer Network Information Center, Chinese Academy of Sciences. The satellite track of path 120 and row 38 gives full coverage of the study area. In order to reduce the influence of clouds on information extraction, relatively cloud-free images were chosen. Further, to minimize the impact water has on the river islands, only images during the dry season were used.

#### 2.2.2. Hydrological Data

The Datong hydrological station, which lies upstream of the study area in the stem stream of Yangtze River, is located in Chizhou City, Anhui. It is an important hydrological station as it is responsible for controlling water flow from the Yangtze River to the sea and protecting water resources. Datong Station has measured water levels, annual runoff, sediment discharge, average sediment concentration, among other parameters, since 1950. Based on statistics from the Jiangsu Province Water Resources Bulletin [19], China River Sediment Bulletin [20] and Changjiang Sediment Bulletin [21], the measured average annual runoff for 66 years from 1950 to 2015 is 893.1 billion m^3^, the average annual sediment discharge for 65 years from 1951 to 2015 is 0.368 billion tons, and the annual average sediment concentration for 65 years from 1951 to 2015 is 0.414 kg/m^3^.

### 2.3. Image Preprocessing

**Figure 2:**
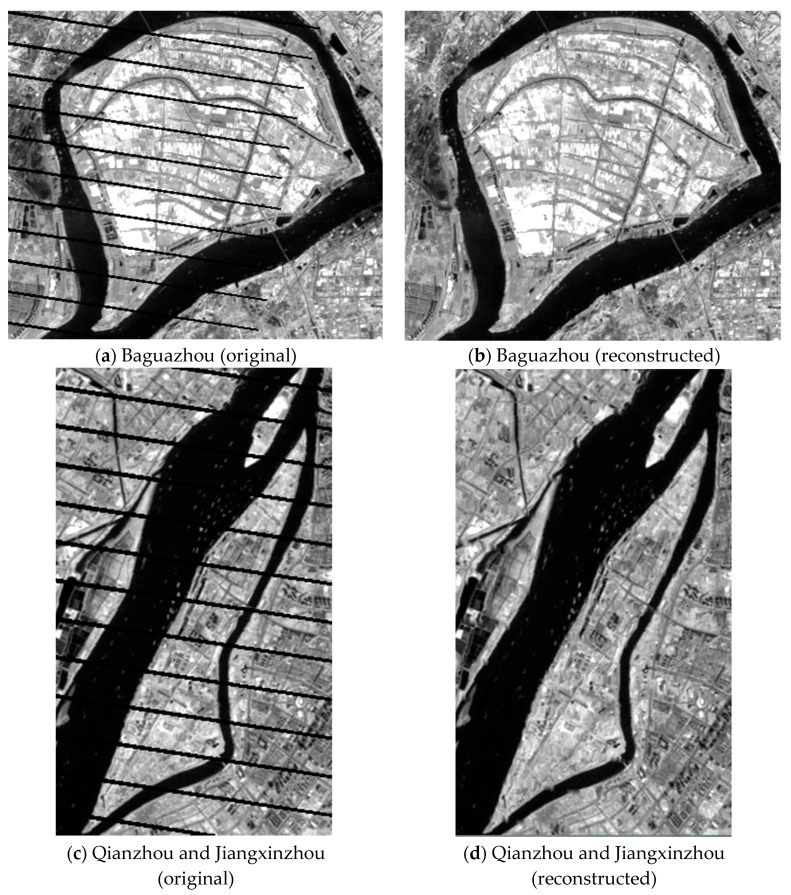
Sample Landsat 7 ETM+ SLC-off model correction effectiveness of 19 December 2005, images over (**a**,**b**) Baguazhou island and (**c**,**d**) Qianzhou and Jiangxinzhou islands.

It is important to note that the Scan Lines Corrector (SLC) of Landsat7 ETM+ on 31 May 2003 suffered a sudden malfunction, which resulted in some images being covered by black stripes over 25%. The SLC-off model was used to rework these images by revising the single band stripe (Figure 2). This paper used the function “tm_destripe” in the ENVI (The Environment for Visualizing Images) image processing software package for this. The basis of tm_destripe is the Multiple Image Local Adaptive Regression Analysis Model or Multiple Image Fixed Window Regression Analysis Model, which can be used to revise stripes. This method can more accurately repair images and hence, can recover surface feature information. It facilitates the interpretation and extraction of information from images and meets present research demands.

The selected TM and ETM+ data in this paper are L1T standard topographic correction products. The products’ geodetic correction relies on precision ground control points and high-precision Digital Elevation Model (DEM) data. The map of the study area can be derived using the band combination and Region of Interest (ROI) subset. The data’s original projection, UTM (Universal Transverse Mercator)-WGS (World Geodetic System) 84 Antarctica Polar Projection, was transformed into an equal area projection, i.e., the Sample Lambert Azimuthal Equal Area Projection. The near-infrared band wavelength of TM and ETM+ is between 0.76 and 0.90 μm, which can be used to effectively distinguish water bodies from vegetation cover. According to field investigations, the main ground object type on the islands is vegetation. Since the near-infrared band can meet the demands of this research, the fourth band of TM and ETM+ were used in this paper. Geometric registration was applied to the near-infrared images of seven years by Ground Control Points (GCPs) and using ENVI 5.1 software (ITT Visual Information Solutions Company, Boulder, CO, USA). These images were cropped to the same ROI.

## 3. Methods

### 3.1. River IslandArea Extraction Methods

TM and ETM+ images are gray-scale images. Each pixel of gray-scale images is described by a quantized discrete gray value [22]. Gray value is also called digital number (DN). DN value is between 0 and 255 because of eight-bit quantization. The DN value of 0 shows black and that of 255 shows white. The reflectivity of rivers, lakes and other water bodies are lower than other surface features, and their DN values are different. Then a binary model was used to distinguish river islands from the Yangtze River water. Building abinarization model in ENVI,

(1) $$\mu =\left\{ \begin{array}{ll} 1 & if\;DN\geq \lambda \\ 0 & otherwise \end{array}\right.$$

where, μ is the image after binarization, DN is the gray value of every pixel in the image, λ is the threshold value of island and the Yangtze River.

**Figure 3:**
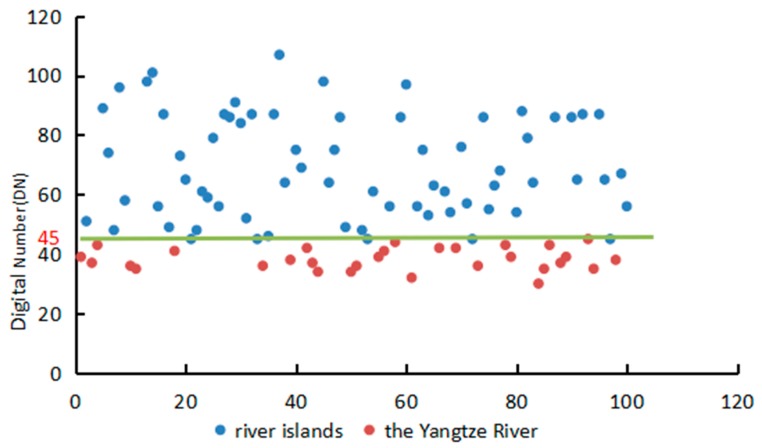
The value of the demarcation between river islands and the Yangtze River. X-axis represents point numbers and y-axis represents digital number (DN) value at this point in the image.

To ensure the value of λ, this study used the threshold value method, which applied the difference in the reflectivity of some bands between a surface feature and other background surface features. According to a priori knowledge of visual image interpretation and field confirmation, we randomly and uniformly selected 100 points from one image and judged whether they are land or not. Then we obtained an optimal threshold value of 45, which is the DN value of water and land demarcation (Figure 3). The threshold value method has a high effectiveness to extract water information based on the significant difference in the reflectivity between water and other objects.

In the process of acquisition and transmission, the image influenced by sensors and atmosphere produces noise. Low-pass filtering (LPF) can filter out high-frequency components and noise, achieving sharp noise removal and image smoothing. Mean value smoothing is a common method of LPF. When the area is given by M×N, the formula is

(2) $$r(i,j)=\sum_{m=1}^{M}\sum_{n=1}^{N}\phi (m,n)t(m,n)$$

where, M×N is the size of the assumed template, r(i,j) is the image after the process of LPF, t(m,n) is the template, ϕ(m,n) is the window which is as the same size as template, and the DN values of pixels in the window are chosen from the image to multiply t(m,n) t(m,n) is actually a convolution kernel. Conduct convolution using a 3 × 3 template gives

(3) $$t(m,n)=\left(\begin{array}{ccc} \frac{1}{8} & \frac{1}{8} & \frac{1}{8}\\ \frac{1}{8} & 0 & \frac{1}{8}\\ \frac{1}{8} & \frac{1}{8} & \frac{1}{8} \end{array}\right)$$

To filter out noise, 3 × 3 template is enough and the templates bigger than 3 × 3 will cause the loss of information. The usage of t(m,n) puts first data point at center pixel eroding edges slightly. Unsupervised classification was conducted by the K-means method. The basis of K-means is to minimize the distance square sum between multi-mode points and the category center point and move various category center points successively by iteration until it comes out with an optimal result.

After classifying, the images were discontinuous in space and spots or empty areas were formed in the classification regions. It was therefore necessary to conduct reclassification for these small spots. Further cluster analysis was carried out on the results of classification by using the Clump Class function in ENVI and by merging the adjacent similar classification regions using mathematical morphological operators. This process is different from LPF, because the classification information of images after smoothing with LPF was disturbed by adjacent classification encoding. However, with cluster analysis this deleterious effect was not produced. Then continuous images were obtained for further calculation.

At last, vectorization of the images was employed by ENVI, and calculation of the aforementioned seven island areas was done using the Geometry Calculator function in ArcGIS software.

### 3.2. River Island Area Trend Methods

The shape and area information of selected river islands during 1985–2015 were determined through a series of remotely sensed image processing. To understand the area change trend clearly, the anomaly of each island’s area and the total area of seven islands respectively was calculated using the formula:

(4) $$\Delta A_{ik}=A_{ik}-{\bar{A}}_{k}$$

where, k refers to a particular island or islands, i refers to a particular year, $\Delta A_{ik}$ is the area anomaly of a particular island in a particular year, $A_{ik}$ is the area of a particular island in a particular year, ${\bar{A}}_{k}$ is the 30-year average area of the particular island or islands.

Based on the areas of 1985, the formula of average area change rates derived as:

(5) $$\eta_{12}=\frac{A_{2}-A_{1}}{5\times A_{1}}\times 1000‰$$

where, $\eta_{12}$ is the annual average area change rate in a time period and the dimension of η is 1/year, $A_{1}$ is the island’s former size and $A_{2}$ is the island’s latter size, “5”in the denominator is 5 years. When $\eta_{12}$ is greater than zero it indicates area increase, zero indicates no change, and negative indicates area decrease. The annual average area change rate during the 30 years is the mean value of $\eta_{12}$ in the six-time periods.

### 3.3. Analytical Methods of Influencing Factors 

The correlation between the area of all seven river islands sand influencing factors including annual runoff and sediment discharge was derived using Pearson’s correlation coefficient, based on pixel space method with the unit of year. The formula is:

(6) $$R_{xy}=\frac{\sum_{i=1}^{n}\left[\left(x_{i}-\bar{x}\right)\left(y_{i}-\bar{y}\right)\right]}{\sqrt{\sum_{i=1}^{n}{\left(x_{i}-\bar{x}\right)}^{2}\sum_{i=1}^{n}{\left(y_{i}-\bar{y}\right)}^{2}}}$$

where, $R_{xy}$ is the correlation coefficient, $x_{i}$ is the area of river islands in a given year and $y_{i}$ is the influencing factor in a given year, $\bar{x}$ is the 30-year average area of river islands, y¯ is the 30-year average value of influencing factors and i is the particular year. $R_{xy}$ can reflect the close relations of area changes and influencing factors. If $R_{xy}$ is greater than zero, the correlation is positive, otherwise correlation is negative. The closer the value of $\left|R_{xy}\right|$ is to 1, the higher the correlation. In general, the degree of correlation is divided into four classes as follows (Table 2):

## 4. Results

### 4.1. River Island Area Extraction

**Figure 4:**
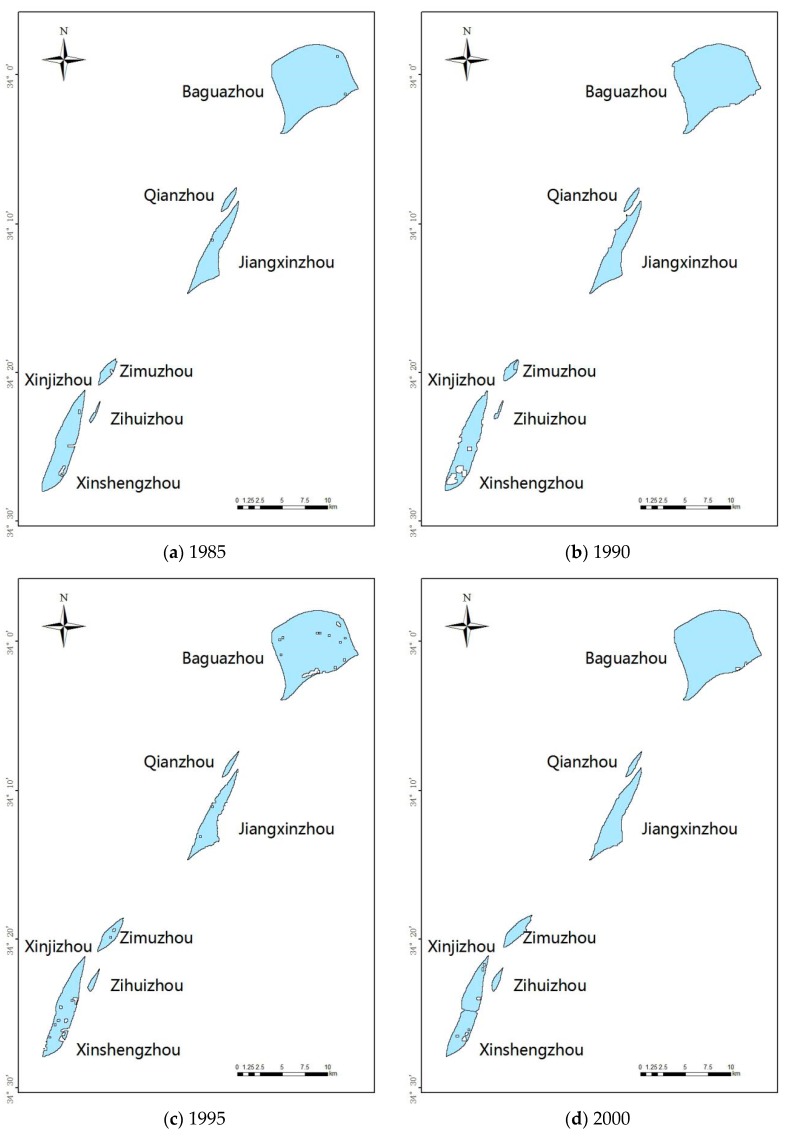

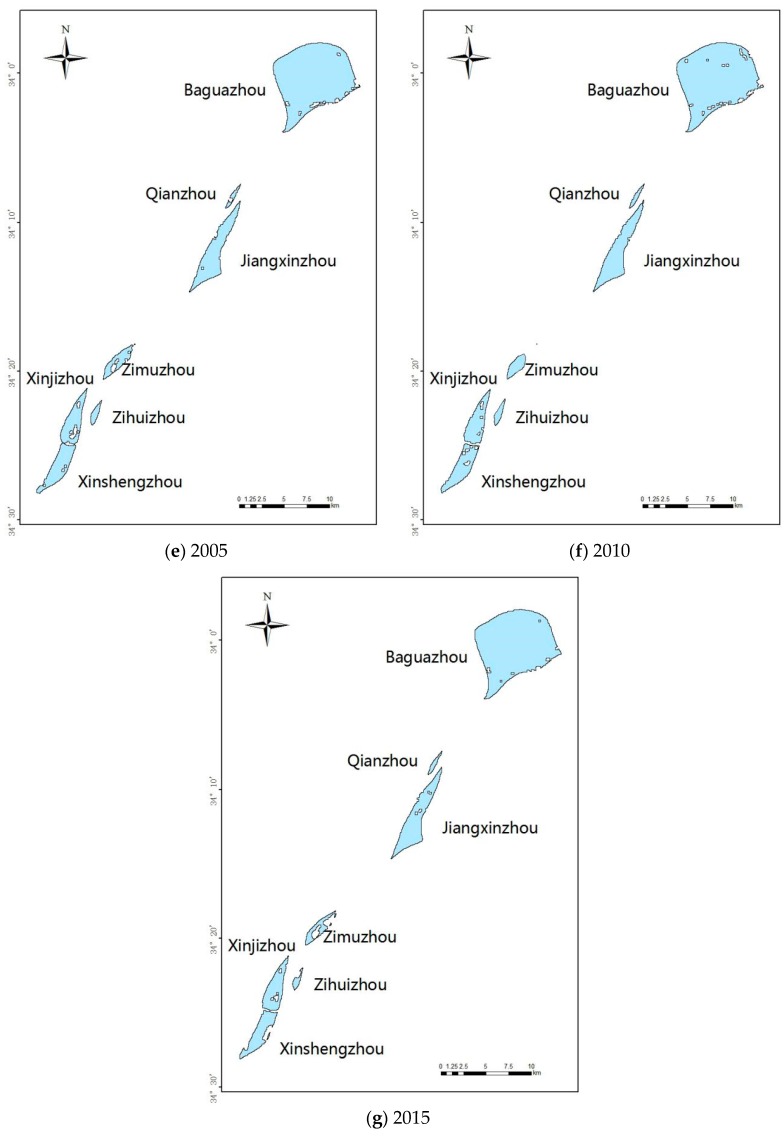
River island changes in NYR during 1985–2015 (**a**–**g**).

**Figure 5:**
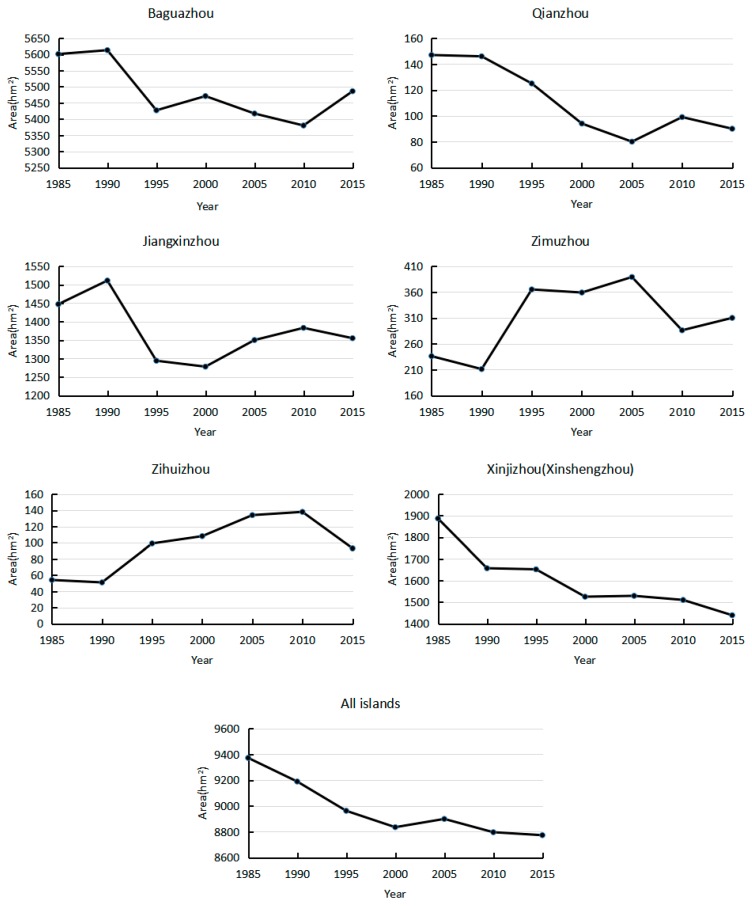
Area changes of river islands in NYR (Nanjing section of the Yangtze River) during 1985–2015 (hm^2^).

The images of river islands obtained after classifying and clustering are shown in Figure 4. Figure 5 is the calculation of these islands’ areas during 1985–2000. The largest island, Baguazhou, and the smallest island, Zihuizhou, have an average size of 5485 hm^2^ and 96.71 hm^2^ (1 hm^2^ = 10,000 m^2^), respectively. The islands listed in order of size, from largest to smallest were: Baguazhou, Xinjizhou (Xinshengzhou), Jiangxinzhou, Zimuzhou, Qianzhou and Zihuizhou. It should be noted that Xinjizhou and Xinshengzhou were linked together but divided into two river islands in 1998. So the areas of these two islands were grouped together for calculations.

### 4.2. River Island Area Evolution Trend

**Figure 6:**
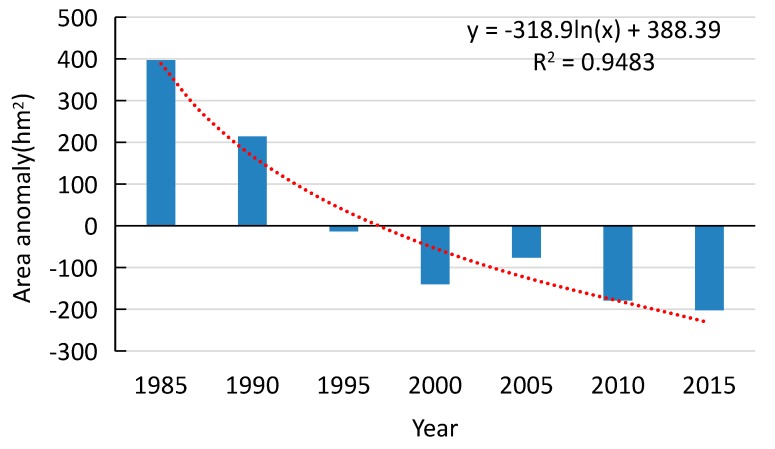
Area anomaly of the seven river islands in the study area (hm^2^).

**Figure 7:**
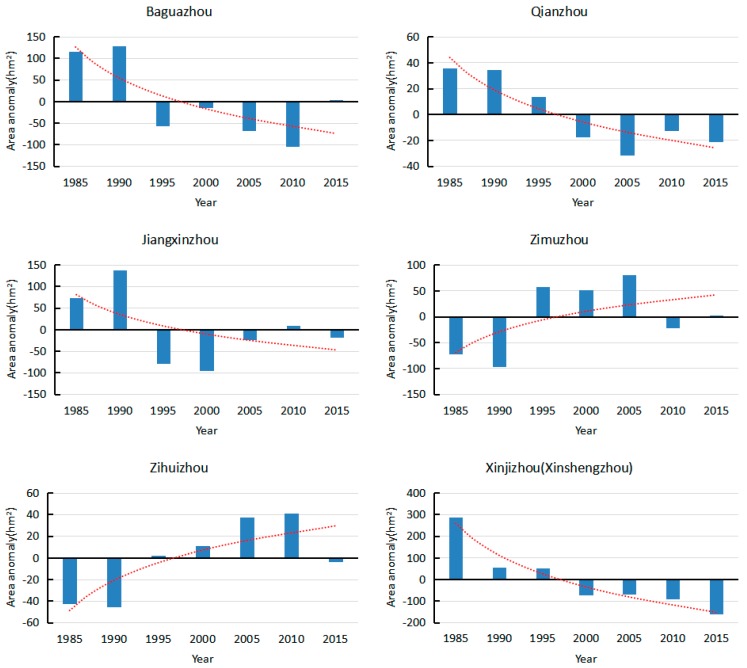
Area anomaly of each island in the study area (hm^2^).

The trend of all islands’ area and each island’s area were calculated by Formula (4) respectively. The total area of the river islands in NYR clearly decreased during the 30 years (Figure 6). The area decreased faster during 1985–2000 and slower from 2000 to 2015. These islands presented a general decrease with a 30-year annual average area of 8975.14 hm^2^. The total area declined by an average of 19.97 hm^2^ a year. Every island’s area change can be seen clearly in Figure 7. The areas of Baguazhou, Qianzhou, Jiangxinzhou and Xinjizhou (Xinshengzhou) islands decreased, and that of Zimuzhou and Zihuizhou islands increased during the 30 years.

**Figure 8:**
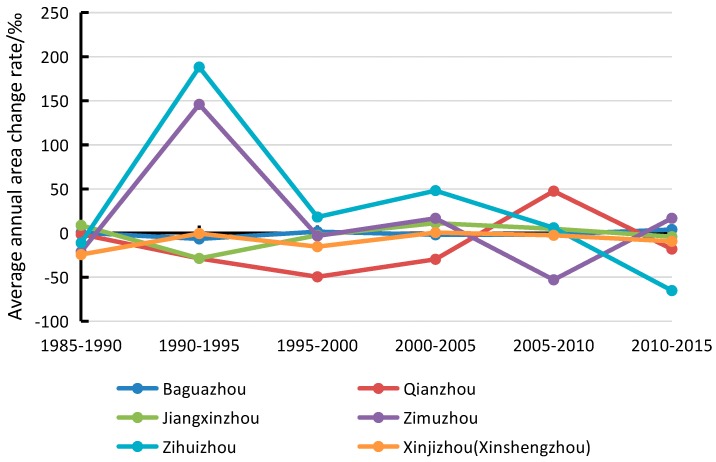
Average annual area change rate of the islands in the study area (‰).

The annual average area change rate during the six-time periods are shown in Figure 8 by Equation (5). The largest magnitude of rate of area change occurred with Zihuizhou, which increased by 30.7‰ per year. The smallest island, Baguazhou, decreased by 0.66‰ per year. Listing the islands from largest to smallest, in order of the absolute value of each island area change rate were: Zihuizhou, Zimuzhou, Qianzhou, Xinjizhou (Xinshengzhou), Jiangxinzhou and Baguazhou. Disregarding Zihuizhou, each island area change rate tended towards zero, with time, suggesting a trend towards stability.

### 4.3.CorrelationAnalysisof Influencing Factors

**Figure 9:**
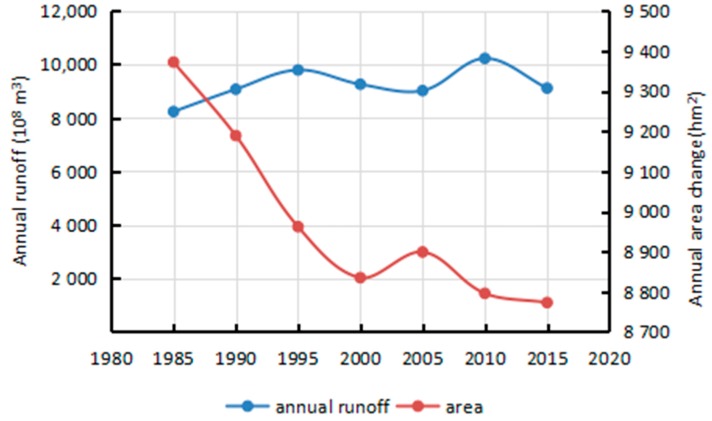
Changes of annual runoff and area.

**Figure 10:**
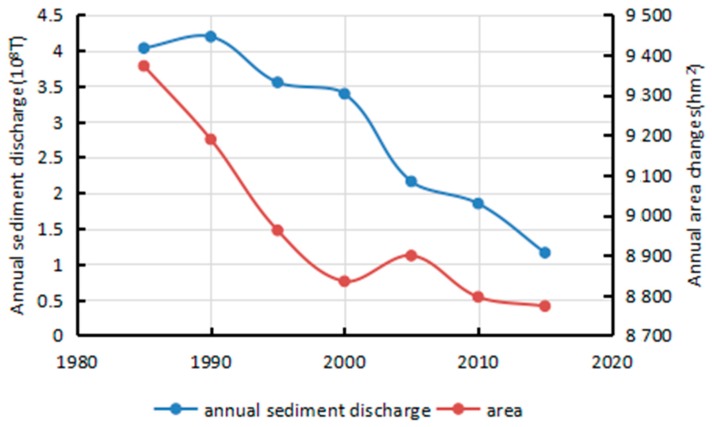
Changes of annual sediment discharge and area.

The results of the correlation analysis conducted on 7 selected years’ runoff and sediment discharge data are displayed in Figure 9 and Figure 10. The results indicate that river island areas are inversely proportional to annual runoff and proportional to annual sediment discharge, with a correlation coefficient of −0.68 and 0.79, respectively. Within a certain range, the area exhibited a small decrease with the increase in runoff and a larger decreased with the drop-in sediment discharge. The change in area was noticeable due to three great floods which occurred during that period. Consequently, the area was more affected by sediment discharge than runoff. Drastic reduction of sediment discharge preceded the lack of sediment particles which are important to island’s growth. Especially, since many big dams and reservoirs were built in the upper-stream, there was a direct and drastic reduction of sediment discharge and concentration. On one hand was the continual washing of relatively clean water, and on the other hand was no net import of sediment, which led to a negative growth of the islands.

## 5. Discussion

### 5.1. Description of the Islands and Analysis of River Island Area Changes

Neglecting Xinjizhou and Xinshengzhou, which were originally only one island, the largest islands were Baguazhou and Jiangxinzhou. Concerning their own areas, very little change was observed during the past 30 years. The area of these islands decreased slightly, but were relatively, remained stable.

Baguazhou, located in the northwest of Qixia District, Nanjing City, has a plain-type sandbank classically formed by scoured and accumulated silt. The island is low and flat and its northwest part is a slightly higher than its southeast area. Its ground elevation is between 5.2 and 7.7 m (Wusong Elevation) but mostly under 6.5 m. The part above 6.5 m is found along Xiaojiang River in the northwest. Established islands lie at a higher elevation than building and pioneer islands [23]. Upon reaching Baguazhou, the Yangtze River split into two channels, south and north. The south channel, being the main current stream, had a maximum depth of 35 m. The northern channel, which is a tributary, had a maximum depth of 10 m. A primary and secondary flood banks were built around the island. Most of the secondary flood bank was along the periphery and protected the island from general flooding. The top mark of the primary flood bank’s anti-flood wall reached up to 12.5 m and it has the ability to control the worst flooding in a century. Hence, flood banks play important roles in the stability of Baguazhou.

Jiangxinzhou, located to the west of Jianye District, Nanjing City, runs in a southwest to northeast direction. From north to south, Jiangxinzhou has a length of 12 km, from east to west, an average of 1.2 km and it had an average area of 1406.86 hm^2^ over the past 30 years. It was formed by sediment erosion and deposition with its current contour having taken form during the Song Dynasty. Also called Plum Island because of its lanky plum looking shape, Jiangxinzhou holds an important position in development along and across the Yangtze River. Since the Qing Dynasty when the island was opened up for development, Jiangxinzhou has now an established agriculture and tourism industry. Consequently, its shore experienced a high degree of development, factors which greatly affected its surface area.

Qianzhou is to the east of Yangtze River’s main channel in Jianye District. It has a 30-year average area of 111.57 hm^2^ which decreased on average by 13.37‰ per year. Most area of the island is still in its natural state, covered in fields of wild reed and desolate beaches. The land above water level shows distinct seasonal impacts. Since Qianzhou is off the beaten track, anthropogenic influences on the island have been minimal, with little change in area.

**Figure 11:**
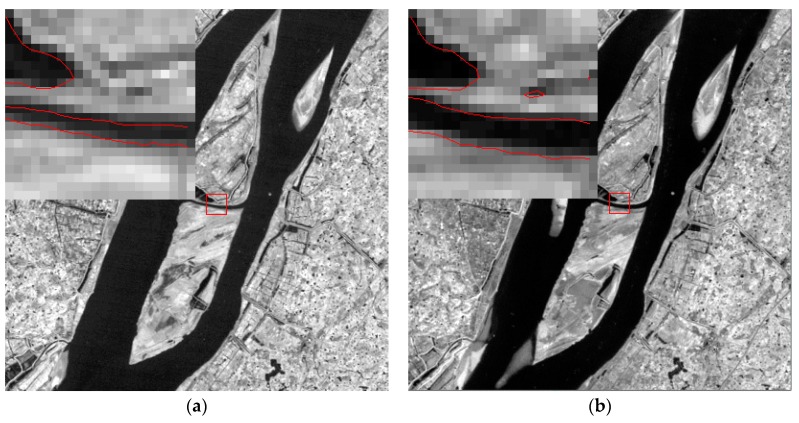
Before-and-after images of mid-channel between Xinjizhou and Xinshengzhou ((**a**) 7 February1998; (**b**) 19 December1999).

Xinjizhou (Xinshengzhou) has an average area of 1599.86 hm^2^ and it has shrunk by 448 hm^2^ in the past 30 years, decreasing on average by 8.62‰ a year. Xinjizhou and Xinshengzhou are located in the upstream of the Jiangsu section along the Yangtze River. A typical bottomland island with its southern bank belonging to Jiangning District and the northern bank belonging to Pukou District, Xinjizhou and Xinshengzhou were once linked together. During the early 80s when Yangtze River water potential changed, a ditch of more than ten meters wide was dug, which resulted in the splitting of one island into two. After a catastrophic flood within the Yangtze Basin in 1998, people on the island were forced to withdraw from the area. The flood water was so great that the ditch expanded to a mid-channel of more than 2000 m long, 200 m wide, and 10 to 14 m deep. The change of the mid-channel before and after the flood can been clearly seen in the images of 1998 and 1999 by automatic contour-line extraction method (Figure 11). At present, the mid-channel is still evolving and its width is increasing every year. The stability of the islands and the dikes along the banks of Yangtze River will be affected. The security of the main channel of Yangtze River will be threatened. Collapse and washing are the primary causes of these islands’ area decrease. A few remediation projects over the years have slowed down the rate of erosion and even led to some measure of recovery.

### 5.2. Analysis of River Island Shape Changes

**Figure 12:**
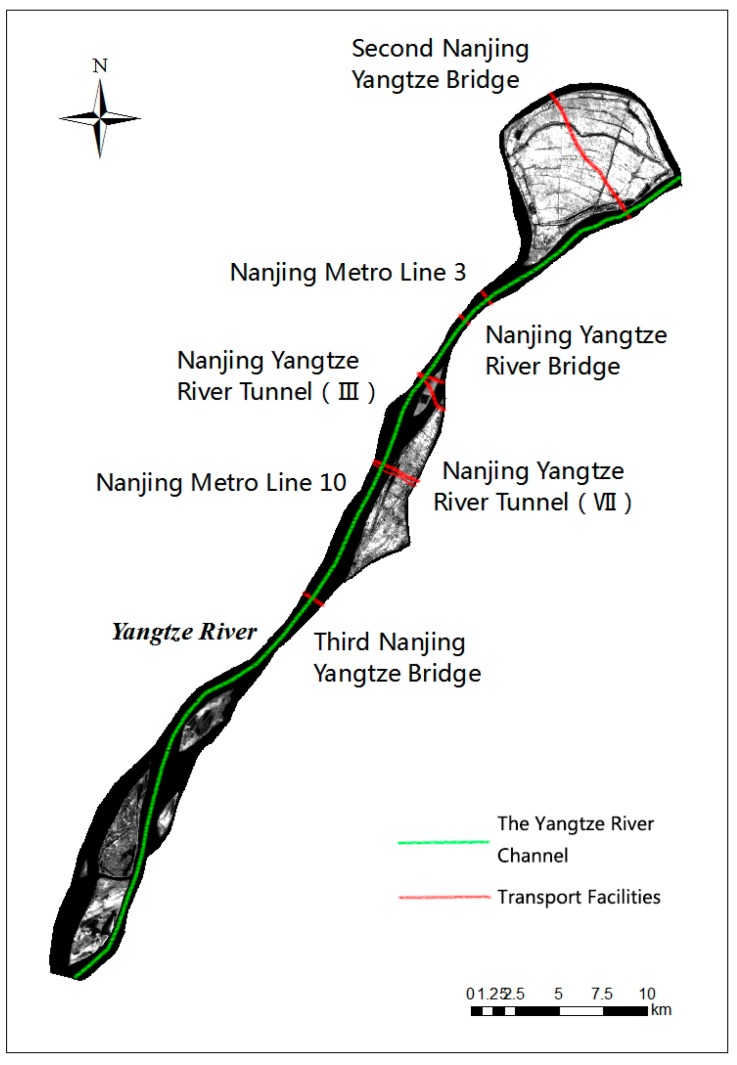
Map of the channel in NYR by band 4, Landsat 7 ETM+ acquired on 10 October 2000.

By comparing TM and ETM+ images at different times, changes on the islands’ shapes were distinctly visible. The shape changes of the larger islands like Jiangxinzhou and Baguazhou were not very obvious. On the hand, smaller islands like Zimuzhou and Qianzhou displayed large transformation as their heads eroded and vanished and their ends grew due to sediment deposits, thus moving the entire body downstream. Due to the inflexion point around Baguazhou, the river current was weaker which resulted in a weakened scouring effect. The remaining six islands were all long and narrow, and the protuberant banks trended toward smoothness because of long-term washing. Most islands are located in the east of the channel (Figure 12). The split ratio of the western channel is greater than the eastern channel, allowing more water to flow on the west side. Ships traveling along the west side caused waves lapping against the shoal and intensified the erosion of western banks. This led to these islands’ shapes to shift more on the western bank than on the eastern banks.

### 5.3. Factors Influencing of River Island Changes

#### 5.3.1. Natural Factors

**Figure 13:**
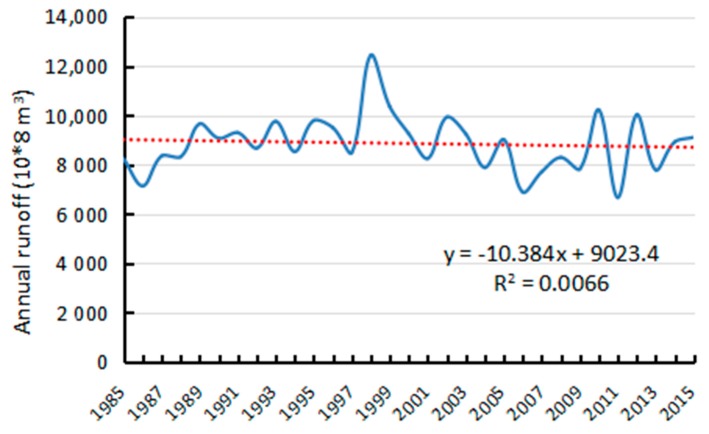
Annual runoff of Datong hydrological station (10^8^ m^3^).

**Figure 14:**
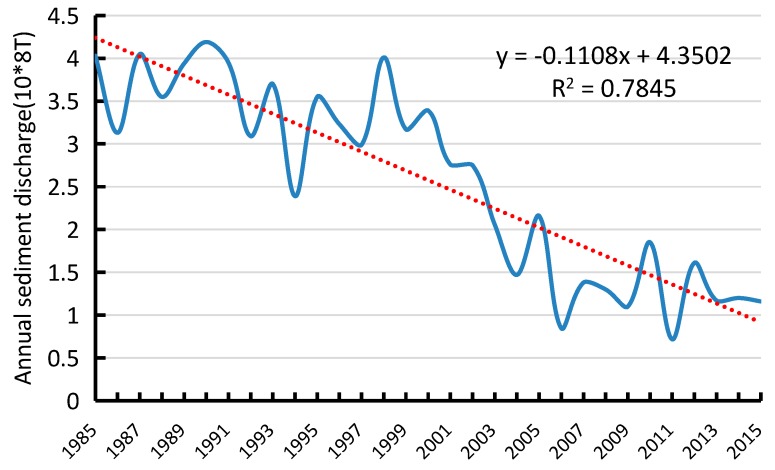
Annual sediment discharge of Datong hydrological station (10^8^ T).

Rivers with high sediment loads experience annual migration rates that are higher than those of rivers with lower sediment loads [24]. This study processed the annual runoff and sediment discharge data during the period 1985–2015 as measured by the Datong hydrological station (Figure 13 and Figure 14). The average annual runoff in the 30 years was measured at 8857.29 × 10^8^ m^3^ with a small decreased rate of 10.384 × 10^8^ m^3^ per year. The maximum runoff of 10,354.77 × 10^8^ m^3^ and minimum runoff of 6671 × 10^8^ m^3^ were recorded in 1999 and 2011, respectively. The average annual sediment discharge in the 30 years was 2.62 × 10^8^ T and decreased drastically by 0.1108 × 10^8^ T per year. The maximum sediment discharge of 4.19 × 10^8^ T was recorded in 1990, whereas the minimum sediment discharge of 0.718 × 10^8^ T was measured in 2011. Associating with correlation analysis of influencing factors, sediment discharge is the main cause for the area decrease of river islands.

Apart from flow-sediment conditions, meteorological factors such as precipitation [25,26,27], natural factors such as vegetation coverage and vegetation type [28,29] and climatic factors such as the monsoon, also influenced the evolution of river islands to a considerable degree. During times of heavy precipitation, water in the lakes of an island will spill over and flow into Yangtze River, scouring as it goes, forcing the loss of soil and shoals. However, as is apparent, vegetation serves a remarkable function of water and soil retention. Comparatively, arbor is better than shrubbery and sod, mixed forest is better than pure forest in this respect. Good vegetation type and coverage can promote the stability of river islands.

#### 5.3.2. Human Factors

With socio-economic development, anthropogenic influence plays an increasingly significant role. Banks collapse caused by over-exploitation and unreasonable land use always happens. It threatens the safety of the channel and influences the evolution of river islands. For example, since 2011, the Elwha River dam in Washington State which stored about 21 million m^3^ of sediment was removed gradually, and geomorphic evolution, ecosystem and environment along the river were deeply affected by the project [30]. More than 5000 reservoirs have been constructed along the Yangtze River basin since the 1950s [31], and the biggest key water control project in human history, the Three Gorges Reservoir, started to store water in 2003 when the water level in front of the dam was 135 m. In October 2010, the level reached 175 m for the first time, which was the normal water level. According to the Changjiang Sediment Bulletin, sediment deposited in the Three Gorges Reservoir reached 0.449 × 10^8^ T in 2014. The sediment delivery ratio of the reservoir was 19.0%. Thus, a strong link between the Three Gorges Reservoir and the great decrease of sediment discharge in the lower reaches of Yangtze River can be found. In addition, river channel sand mining activities in the middle and lower reaches also affected sediment transport [32,33].

A number of soil and water conservation projects have been conducted in Jiangsu Province and upstream Anhui Province in recent years. In 2013, NDRC (National Development and Reform Commission) approved a total investment of 7.21 million USD for the regulation project of Xinjizhou in NYR. The project is aimed at plugging the mid-channel between Xinjizhou and Xinshengzhou, thus curbing the trend of south channel’s increasing split ratio. It will weaken scouring in the mid-channel, and protect river channels and dikes along the two banks.

## 6. Conclusions

Applying TM and ETM+ images of Nanjing City, this study takes advantage of threshold value method, binarization model and a series of image processing techniques to extract information about the areal changes of seven riverine islands in the lower reach of the Yangtze River over 30 years, split into seven 5 year time periods. The characteristics of their evolution and influencing factors were then analyzed. The conclusions are as follows:

(1) Landsat TM and ETM+ images can meet the requirements to study ground features at certain time scales and spatial resolutions. They have good adaptability in extracting information from water and land area such as river islands. ETM+ images reconstructed by the SLC-off model can also meet research criteria.
(2) The total area of the seven river islands in NYR exhibited a continuous decrease of 19.97 hm^2^ per year. However, a more rapid decrease of 25.8 hm^2^ per year was observed during the period 1985–2000, while a slower decrease of 4.13 hm^2^ per year was noted during the 2000–2015 period. The largest absolute value of the rate of area change was at Zihuizhou, which increased by 30.7‰ per year whereas the smallest recorded value was at Baguazhou with a recorded decrease of 0.66‰ per year.
(3) Although all seven islands had different degrees of atrophy, the shape changes of the largest islands, Baguazhou and Jiangxinzhou, were not obvious. On the contrary, smaller islands like Zimuzhou and Qianzhou displayed significant changes. The heads of the river islands eroded away, vanishing as their tails grew, resulting in downstream migration. With the exception of Baguazhou, the remaining islands were all long and narrow because of the long-term washing. Their bank lines were all smoothed and the longitudinal axis direction of their bodies laid all along the Yangtze River’s direction. The erosion of the west bank was observed to be more intense than that of the east.
(4) According to correlationanalysis, the island areas are inversely proportional to annual runoff with acorrelationcoefficient of −0.68, and proportional to annual sediment discharge with a correlationcoefficient of 0.79, that is, the area is more affected by sediment discharge than runoff. The evolution of river islands is simultaneously affected by both natural and anthropogenic factors. In particular, human activities changed the imports of exogenous sediment particles and are thus playing increasingly important roles in the evolution of these riverine islands.

## Figures and Tables

**Table 1:** Remote sensing data with the resolution of 30 m used in the study area.

Date	Satellite/Sensor	Center Latitude (°)	Center Longitude (°)
18/November/1985	Landsat5/TM	31.75	118.80
4/December/1990	Landsat5/TM	31.76	118.80
30/January/1995	Landsat5/TM	31.75	118.89
10/October/2000	Landsat5/TM	31.73	118.89
19/December/2005	Landsat7/ETM+	31.73	118.89
17/December/2010	Landsat7/ETM+	31.74	118.86
12/October/2015	Landsat7/ETM+	31.74	118.86

TM: Thematic Mapper; ETM: Enhanced Thematic Mapper.

**Table 2:** Correlation Classification.

$R_{xy}$	$\left|R_{xy}\right|<0.3$	$0.3\leq \left|R_{xy}\right|<0.5$	$0.5\leq \left|R_{xy}\right|<0.8$	$0.8\leq \left|R_{xy}\right|<1$
Degree of correlation	weak	low	moderate	high
